# Gastric glomus tumor: a case report and review of the literature

**DOI:** 10.1186/s13256-021-03011-0

**Published:** 2021-08-16

**Authors:** Eleni S. Tsagkataki, Mathaios E. Flamourakis, Ioannis G. Gkionis, Michail I. Giakoumakis, Georgios N. Delimpaltadakis, Georgios M. Kazamias, Elpida S. Giannikaki, Manousos S. Christodoulakis

**Affiliations:** 1 Department of General Surgery, Venizeleio General Hospital, Leoforos Knossou 44, 71409 Heraklion, Crete Greece; 2 Department of Pathology, Venizeleio General Hospital, Leoforos Knossou 44, Heraklion, Crete Greece

**Keywords:** Gastric glomus tumor, Preoperative diagnosis, Wide local excision, Follow-up

## Abstract

**Introduction:**

Gastric glomus tumor is a rare mesenchymal neoplasm. There are only a few cases of the tumor showing malignancy, and there are no specific guidelines for the management of this entity.

**Case presentation:**

We present the case of a 53-year-old Caucasian male who was hospitalized for anemia. Computerized tomography of the abdomen depicted a mass between the pylorus of the stomach and the first part of the duodenum. Preoperative diagnosis was achieved with pathology examination of the biopsies taken via endoscopic ultrasound and upper gastrointestinal endoscopy. An antrectomy with Roux-en-Y anastomosis and appendicectomy, due to suspicion of appendiceal mucocele, were performed. The patient had an uneventful postoperative recovery and was discharged 5 days later.

**Discussion:**

Preoperative diagnosis of a gastric glomus tumor is difficult owing to the location of the tumor and the lack of specific clinical and endoscopic characteristics. Furthermore, it is exceptional to establish diagnosis with biopsies taken through endoscopic ultrasound or upper gastrointestinal endoscopy, prior to surgical resection. Although most glomus tumors are benign and are not known to metastasize, there are rare examples of glomus tumors exhibiting malignancy. Treatment of choice is considered wide local excision with negative margins. However, long-term follow-up is required as there is the possibility of malignancy.

**Conclusion:**

The aim of this report is to enlighten doctors about this uncommon pathologic entity. Surgical resection is considered the golden standard therapy to establish a diagnosis and evaluate the malignant potential.

## Introduction

Glomus tumor (GT) is a rare mesenchymal neoplasm and accounts for approximately 1% of gastric mesenchymal tumors [1, 2]. Barre and Masson first described the clinical and pathological features of glomus tumor in 1924, and the term gastric glomus tumor (GGT) was first reported in February 1948 [3, 4]. In most cases, the tumor is benign, and only in a few of them does the neoplasm show malignancy [5–8].

The neoplasm arises from the glomus body, which is an arteriovenous anastomosis consisting of tightly convoluted capillaries surrounded by modified muscle cells [9, 10]. These modified muscle cells are known as the glomus cells [9, 10]. Regarding gastrointestinal tract, GT is mostly found in the stomach and especially in the antrum [1, 2]. It arises from the submucosa or muscularis propria of the gastric wall and usually spares the overlying mucosa [9–11]. It projects either into the lumen or into the serosa [9–11].

Gastric glomus tumor (GGT) lacks specific clinical and endoscopic characteristics, which renders it difficult to distinguish from other gastric submucosal neoplasms prior to surgical resection [12–14]. The diagnosis of GGT depends on pathological and immunohistochemical findings of the surgical specimen [12–14]. Establishing diagnosis with biopsies taken by either endoscopic ultrasound (EUS) or upper gastrointestinal endoscopy is exceptional [14–17].

Currently, the main diagnostic modalities for evaluating GGT are endoscopic ultrasound (EUS) and computerized tomography (CT) [17, 18]. The former has as advantage the identification of the origin layer of the tumor, and the latter is better in depicting the characteristics of the tumor [18].

We report the case of a 53-year-old male whose gastric glomus tumor was diagnosed preoperatively with biopsies obtained via upper gastrointestinal endoscopy and EUS.

## Case presentation

We present the case of a 53-year-old Caucasian male who was admitted to the hospital owing to fatigue and black stools. His vital signs on the admission were: temperature  36.8 °C, heart rate 70 beats per minute, respiratory rate 17 breaths per minute, and blood pressure 120/80 mmHg.

The patient was pale but with good nutrition status [body mass index (BMI) 23.1 kg/m^2^]. He did not consume alcohol or tobacco. He was married and had two children aged 15 and 19 years.

He was taking no medication and had no other underlying disease. Moreover, his medical history was free of any previous surgical interventions. During the clinical examination, there was no sensitivity or tenderness in the abdomen and the bowel sounds were normal. Digital rectal examination revealed the presence of black stools. Clinical examination of cardiopulmonary and urogenital systems showed no abnormal signs. Apart from fatigue, there were no other findings on physical and neurological examination.

The blood tests depicted anemia (hemoglobin 6.0 g/dl with normal values between 13.4 and 17.4 g/dl, and hematocrit 20% with normal values between 41% and 53.8%). The results of all other markers were within normal range (Table 1).

**Table 1.** Results of blood test markers during admission

Blood test markers	During the admission	Normal value range
Hb	6.0	13.4–17.4 g/dl
Hct	20	41–53.8%
WBC	8.900	3.800–10.500/mL
PLT	250.000	150,000–400,000/μl
UR	48	15–50 mg/dl
Cr	1.25	0.7–1.3 mg/dl
SGOT	30	5–35 U/L
SGPT	31	0–55 U/L
GGT	48	0–50 U/L
Na	137	136–145 mmol/L
K	4.5	3.5–5.1 mmol/L
CRP	0.02	< 0.5 mg/dl

*Hb* hemoglobin, *Hct* hematocrit, *WBC* white blood cells, *PLT* platelet, *UR* urea, *Cr* creatinine, *SGOT* serum glutamic oxaloacetic transaminase, *SGPT* serum glutamic pyruvic transaminase, *GGT* gamma glutamyl transferase, *Na* sodium, *K* potassium, *CRP* C-reactive protein

During his admission to the hospital, the patient was transfused with 4 units of blood and 1 unit of fresh frozen plasma, and a Computerized Tomography (CT) scan of the abdomen was performed. The CT scan of the abdomen revealed a mass with vague limits (dimensions 6.8 × 5.7 cm) between the pylorus of the stomach and the first part of the duodenum. Around that mass, there were several lymph nodes with diameter up to 1.6 cm. Furthermore, the presence of appendiceal mucocele was indicated as an incidental finding.

**Fig. 1:**
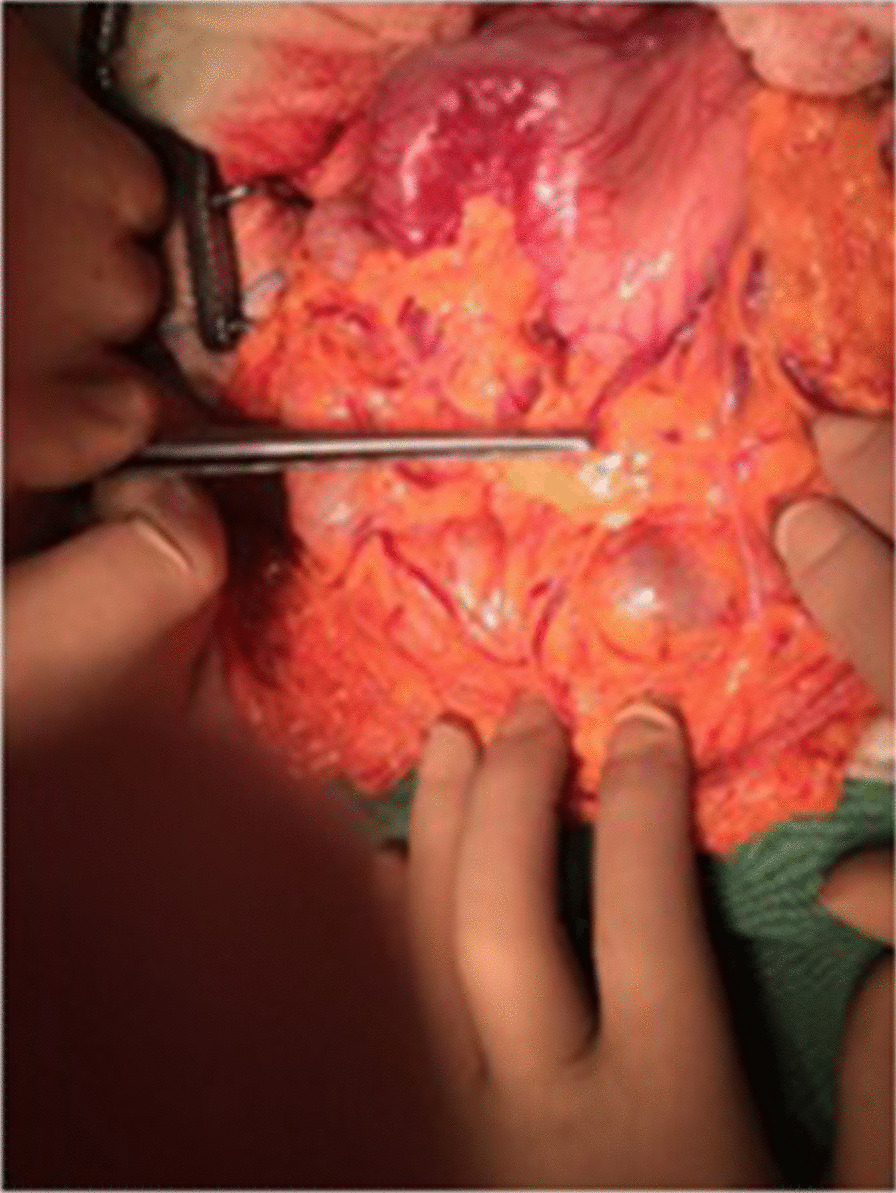
Operative view of gastric glomus tumor

**Fig. 2:**
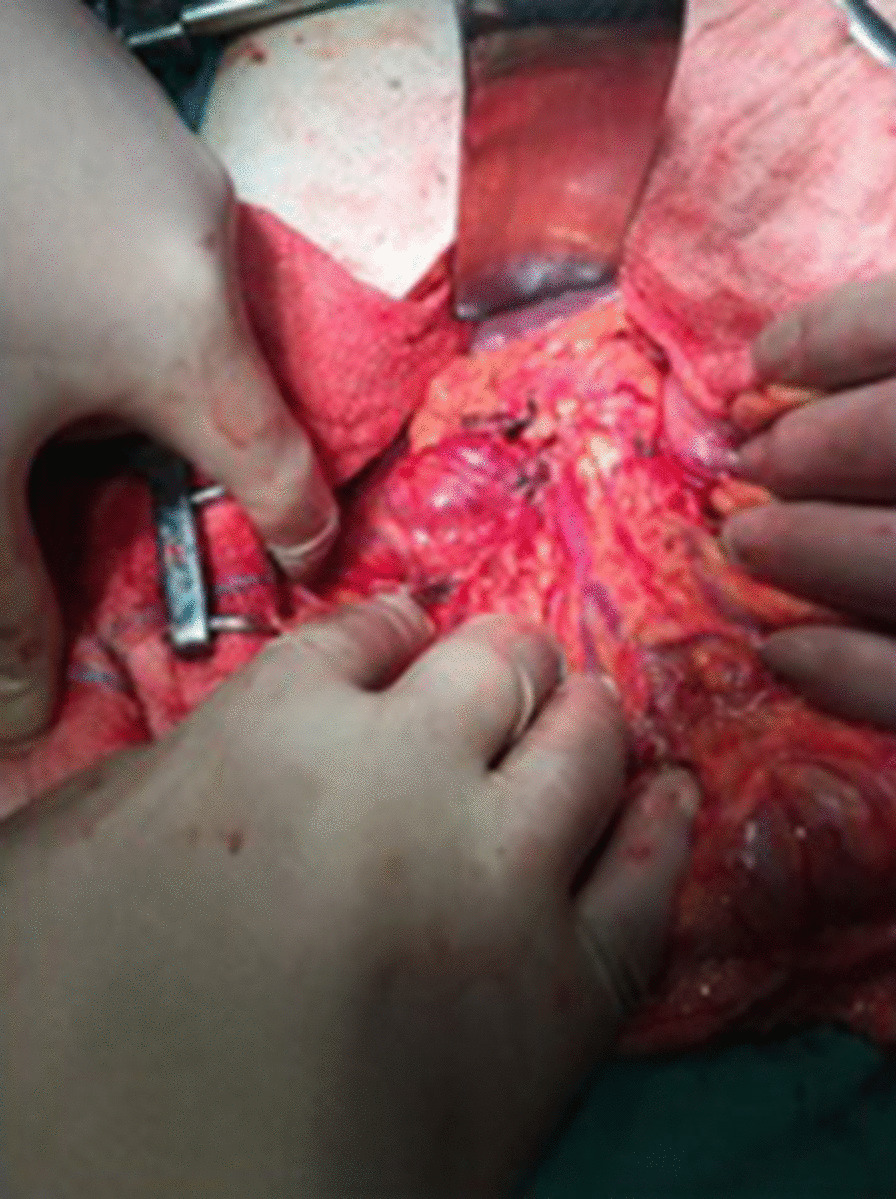
View after antrectomy

On the third day of his hospital stay, the patient underwent EUS and upper gastrointestinal endoscopy, in which the biopsies showed morphologic and immunochemical features compatible with glomus tumor. Twelve days after his admission, the patient underwent open laparotomy with a midline incision. An antrectomy with Roux-en-Y anastomosis and appendicectomy were performed owing to suspicion of appendiceal mucocele from the CT scan (Figs. 1, 2).

The postoperative period was without any incident, and the patient was released in good condition 5 days after the operation. After the surgery and during his hospital stay, he was receiving intravenously 3 g cefoxitin/day for 3 days, 1.5 g metronidazole/day for 3 days, 4 g paracetamol/day for 4 days, and 200 mg tramadol/day for 3 days. The initial postoperatively intravenously administration of fluids was followed by oral feeding after 3 days.

**Fig. 3:**
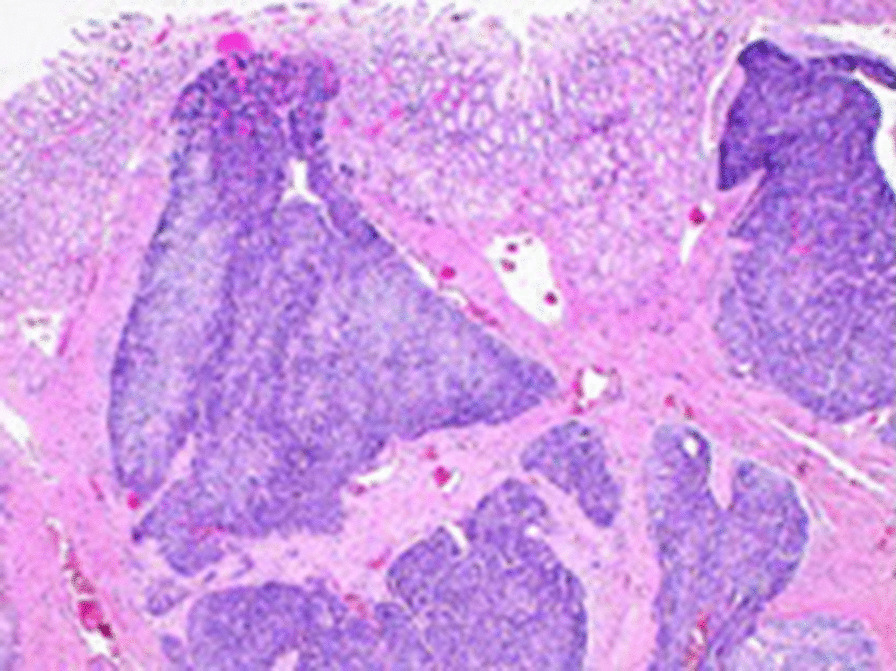
Multiple cellular neoplastic nodules extending into the muscularis propria, submucosa, and gastric mucosa [hematoxylin and eosin (H&E) ×40]

**Fig. 4:**
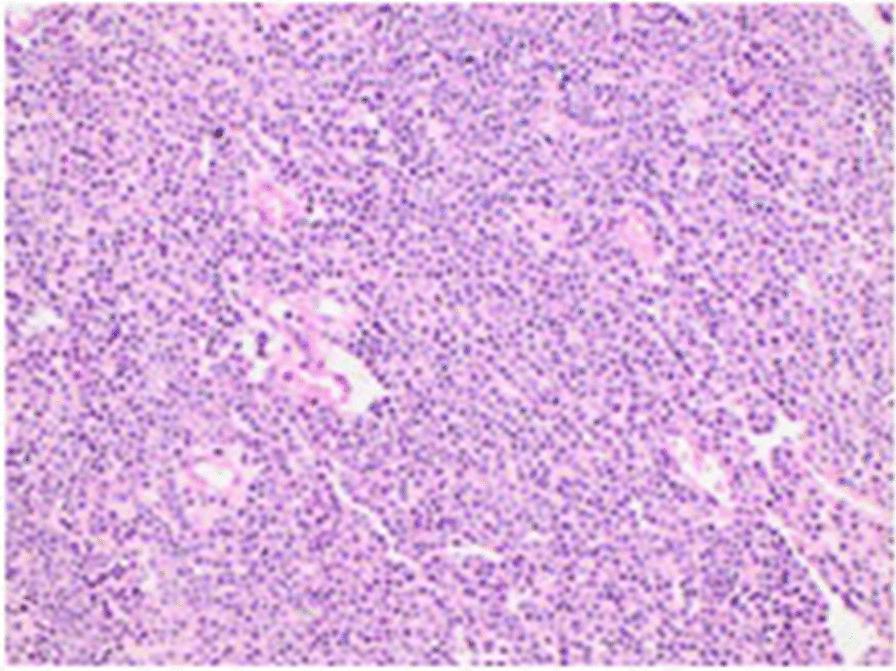
Solid proliferation of cells with round uniform nuclei, light eosinophilic or clear cytoplasm, and highly vascularized stroma (H&E ×200)

**Fig. 5:**
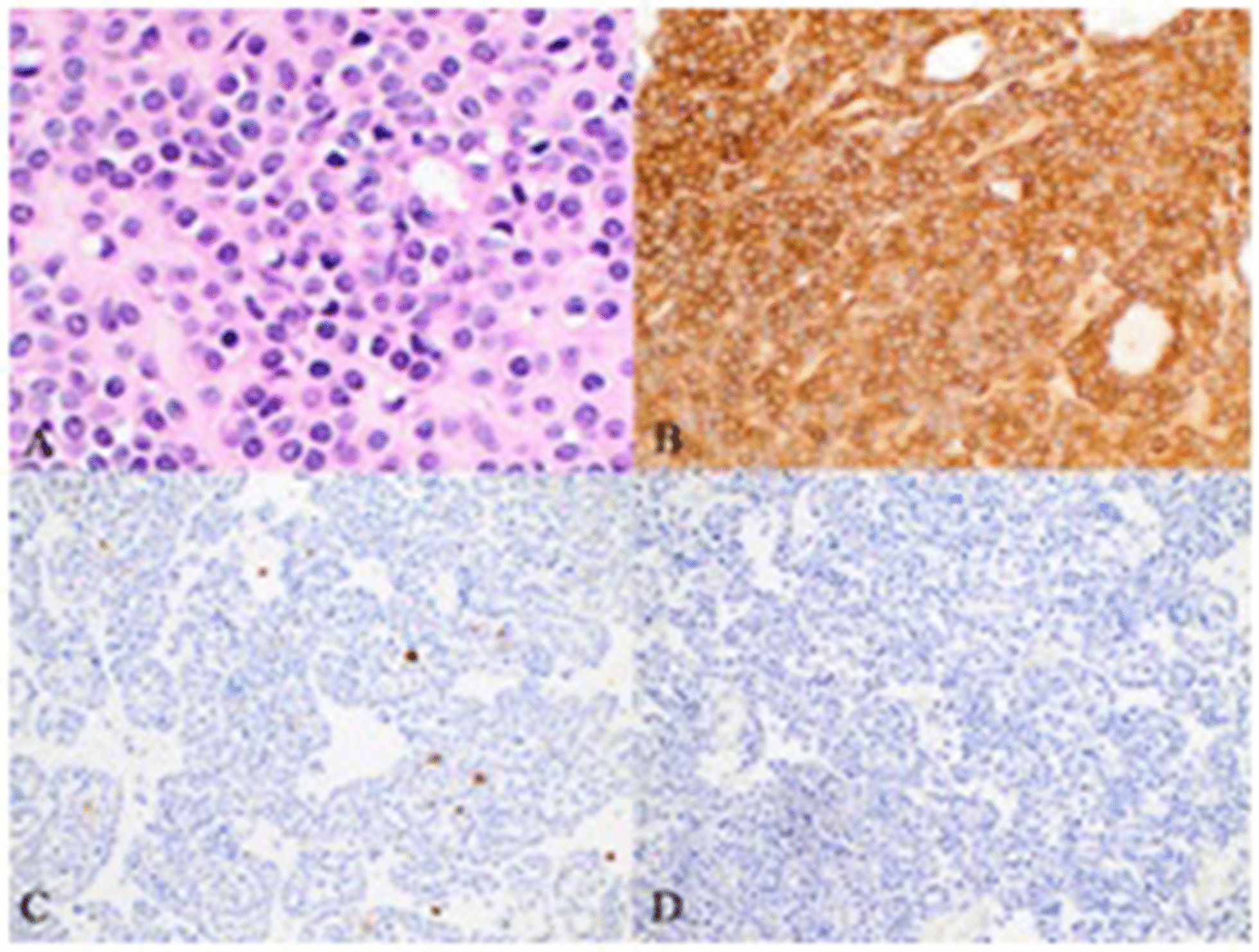
**A** Monotonous cells with well-defined membranes. No mitosis, necrosis, or significant nuclear atypia is observed. Immunohistochemical stains showed diffuse positive cytoplasmic expression of smooth muscle actin (SMA) (**B**), while CD117 (c-kit) (**C**) and desmin (**D**) are negative

Pathology examination showed a 5.5 × 5 × 4.2 cm intramural gastric mesenchymal neoplasm compatible with glomus tumor. Although there was no prominent nuclear atypia, and no mitotic activity or any atypical mitosis, because of the tumor’s size, > 2 cm, and the location, deep in the layers of gastric wall, the final diagnosis was glomus tumor with uncertain malignant potential (Figs. 3, 4, 5). Also, the examination showed appendiceal mucocele with elements of previous rupture.

After discharge from the hospital, the patient underwent regular follow-up meetings with clinical examination, blood tests, and computerized tomography of the brain, the chest, and the abdomen. His last follow-up was in January 2021, 20 months after his admission to the hospital, in which he was found in good clinical status and with no signs of recurrence. There were no findings of pathological entity on the CT of the chest and the abdomen. He was scheduled for a meeting again after 6 months.

## Discussion

We report the case of a rare gastric neoplasm, gastric glomus tumor, in a 53 year-old Caucasian male who was hospitalized for fatigue, black stools, and anemia. There were no pathological findings neither on clinical and physical examination nor on laboratory investigation, apart from black stools in digital rectal examination and signs of anemia in blood tests, but the CT scan of the abdomen that was performed during his admission to the hospital revealed a mass with vague limits between the pylorus of the stomach and the first part of the duodenum. Preoperative diagnosis of GGT is difficult owing to the deep location of the tumor and the lack of specific clinical and endoscopic characteristics [12–14]. It is extremely rare to detect a GGT prior to surgical resection [14–17]. But in our case, the preoperative diagnosis was established with pathologic examination of the biopsies that were taken during the EUS and the upper gastrointestinal endoscopy.

Gastric glomus tumor shares many features with other gastric submucosal lesions, making preoperative diagnosis difficult [12–17]. It is often initially misdiagnosed as gastrointestinal stromal tumor (GIST) [15]. Patients are usually asymptomatic at the time of identification [11, 14]. Clinical symptoms may include epigastric pain, nausea, vomiting, fatigue, ulcerous syndrome, black stools, and, rarely, upper gastrointestinal bleeding [1–4, 19, 20]. In the case of our patient, two of these clinical symptoms were present.

As it can easily be understood, the clinical signs are insufficient to establish the diagnosis of a gastric glomus tumor. Most of these clinical symptoms are common in several pathological entities. Therefore, computerized tomography of the abdomen, EUS, and upper gastrointestinal endoscopy are essential in the diagnosis of a GGT [17, 18]. CT of the abdomen depicts a GT in the stomach as well-circumscribed submucosal mass with homogeneous density on unenhanced images, and strong enhancement on arterial phase images and persistent enhancement on portal venous phase images [21]. Although EUS helps identifying the layer of origin, there are no specific findings in the images that would allow for a convincing preoperative diagnosis [15, 17, 18]. Sometimes heterogeneous echogenicity caused by hemorrhage or calcification may occur [15, 17, 18]. As a conclusion, the images from the EUS only are insufficient to establish a diagnosis [15, 17, 18]. A clinicopathologic analysis showed that diagnostic accuracy can be improved by comprehensive interpretation of the endoscopic and radiologic findings under recognition of this rare but distinctive lesion in the stomach [15].

Due to intramural location, which makes diagnosis with endoscopic biopsy difficult, GT is commonly diagnosed after pathology examination of the surgical specimen [14–17]. However, EUS-guided biopsy has been reported to successfully diagnose gastric glomus tumor in some cases with cytologic and immunochemical analysis of the specimen [16]. However, inadequate acquisition of tissue and the risk of bleeding are major limitations of this technique [16]. Furthermore, the usage of the upper gastrointestinal endoscopy is limited owing to the deep location of the tumor inside the gastric wall [17, 18].

As previously mentioned, GT is commonly diagnosed after pathology examination of the surgical specimen [14–17]. The immunohistochemistry histologic features and the immunohistochemical staining pattern characterize a GT [12–14]. However, biologic behavior cannot be predicted based on histologic appearance, and potential for metastasis cannot be excluded [5–8, 14]. Criteria have been suggested by Folpe *et al*. for defining malignancy in GGT and estimating the risk of recurrence and metastasis [5]. These criteria include the depth, the size (greater than 2 cm), and the combination of high nuclear grade and mitotic activity [> 5/50 high-power fields (HPFs)] [5]. Tumors less than 5.0 cm tend to behave in a benign fashion [5].

Although most glomus tumors are benign and are not known to metastasize, there are rare examples of glomus tumors exhibiting malignancy [5–8, 14]. Folpe *et al*. performed an analysis of 52 cases of glomus tumors located in the extremities and found that 8 of them had histologically confirmed metastases [5]. The most common sites of metastases were the brain, the bones, the small intestine, the lung, and the liver [5]. Song *et al*. reported the case of a malignant gastric glomus tumor with metastases to liver and brain [6]. The histological results of the removed gastric glomus tumor fulfilled the criteria for malignancy proposed by Folpe *et al*. [6].

As far as the surgical operation, treatment of choice is considered the wide local excision with negative margins [14]. There is no need for extended margins of resection or for ample lymph node removal [14]. In our case, due to the position of the tumor, antrectomy with Roux-en-Y anastomosis was performed. Clear margins were obtained, and R0 resection was achieved.

Furthermore, excision of a gastric glomus tumor through EUS has been proposed [17]. However, GGT contains abundant blood vessels, with high risk of intraoperative bleeding, and many hospitals are unaware of this endoscopic treatment technique [17]. Thus, surgical treatment is preferred [14, 17].

As there is a potential for malignancy, long-term follow-up is suggested [14, 20]. Long-term follow-up is required given the rare malignant potential, although no consensus guidelines exist [14, 20]

## Conclusion

Gastric glomus tumor is a rare pathologic entity. It is mainly considered benign and can be cured by surgical resection with excellent outcomes. Some reports present gastric glomus tumors with metastases, which make necessary for a patient carrying a potential malignant GGT to undergo long-term follow-up. Unfortunately, there are no definitive diagnostic criteria or evidence-based treatment guidelines for this entity; thus, it is necessary to have a larger number of cases and studies to acquire the adequate knowledge.

## Data Availability

The datasets generated and analyzed during the current study are available from the corresponding author on reasonable request.

## Abbreviations

GT: Glomus tumor
GGT: Gastric glomus tumor
EUS: Endoscopic ultrasound
CT: Computerized tomography
GIST: Gastrointestinal stromal tumor
